# Prediction of target genes for miR‐140‐5p in pulmonary arterial hypertension using bioinformatics methods

**DOI:** 10.1002/2211-5463.12322

**Published:** 2017-10-21

**Authors:** Fangwei Li, Wenhua Shi, Yixin Wan, Qingting Wang, Wei Feng, Xin Yan, Jian Wang, Limin Chai, Qianqian Zhang, Manxiang Li

**Affiliations:** ^1^ Department of Respiratory Medicine The First Affiliated Hospital of Xi'an Jiaotong University China; ^2^ Department of Respiratory Medicine Lanzhou University Second Hospital China

**Keywords:** GO, KEGG, miR‐140‐5p, target gene, transcription factor

## Abstract

The expression of microRNA (miR)‐140‐5p is known to be reduced in both pulmonary arterial hypertension (PAH) patients and monocrotaline‐induced PAH models in rat. Identification of target genes for miR‐140‐5p with bioinformatics analysis may reveal new pathways and connections in PAH. This study aimed to explore downstream target genes and relevant signaling pathways regulated by miR‐140‐5p to provide theoretical evidences for further researches on role of miR‐140‐5p in PAH. Multiple downstream target genes and upstream transcription factors (TFs) of miR‐140‐5p were predicted in the analysis. Gene ontology (GO) enrichment analysis indicated that downstream target genes of miR‐140‐5p were enriched in many biological processes, such as biological regulation, signal transduction, response to chemical stimulus, stem cell proliferation, cell surface receptor signaling pathways. Kyoto Encyclopedia of Genes and Genome (KEGG) pathway analysis found that downstream target genes were mainly located in Notch, TGF‐beta, PI3K/Akt, and Hippo signaling pathway. According to TF–miRNA–mRNA network, the important downstream target genes of miR‐140‐5p were PPI, TGF‐betaR1, smad4, JAG1, ADAM10, FGF9, PDGFRA, VEGFA, LAMC1, TLR4, and CREB. After thoroughly reviewing published literature, we found that 23 target genes and seven signaling pathways were truly inhibited by miR‐140‐5p in various tissues or cells; most of these verified targets were in accordance with our present prediction. Other predicted targets still need further verification *in vivo* and *in vitro*.

**Abbreviations**
GO: gene ontology
KEGG: kyoto encyclopedia of genes and genome
PAH: pulmonary arterial hypertension
PASMC: pulmonary arterial smooth muscle cell
TF: transcription factor

Pulmonary arterial hypertension (PAH) is a chronic progressive disease of pulmonary vasculature characterized by sustained elevation of pulmonary vascular resistance and pulmonary arterial pressure, consequently leading to right heart failure and eventual death 1. The pathogenesis of PAH is associated with genetic predisposition, inflammation, increase in vascular tone, elevation in pulmonary artery cell proliferation and resistance to apoptosis, and the presence of *in situ* thrombosis 2, 3, 4, 5. Effect of current treatment on PAH remains poor and available therapies to improve long‐term prognosis are limited 6, so exploring novel molecular mechanisms and generating therapeutic approaches are urgently needed.MicroRNAs (miRNAs) are small noncoding RNA molecules around 22 nucleotides long that bind the 3′‐untranslated region (UTR) of mRNA to degrade mRNA and therefore to negatively regulate relevant genes expression 7. miRNAs have the ability to target numerous genes mRNA, therefore potentially controlling a host of genes expression and the activity of multiple signaling pathways 8, 9, 10. Recent studies have shown that reduction in microRNA (miR)‐140‐5p is found in both patients with PAH and monocrotaline‐induced PAH models in rat, which is involved in the development of PAH 11, 12. Therefore, it is important to identify comprehensive downstream targets of miR‐140‐5p with bioinformatics analysis in PAH, and this might provide some critical information for the development and treatment of PAH. In this study, downstream target genes regulated by miR‐140‐5p and upstream transcription factors (TFs) regulating miR‐140‐5p expression were predicted, and the downstream target genes were analyzed for gene ontology (GO) enrichment and Kyoto Encyclopedia of Genes and Genome (KEGG) pathway. Next, the upstream TFs and downstream targets of miR‐140‐5p were determined according to the TF–miRNA–mRNA network. Finally, the direct downstream targets and relevant signaling pathways regulated by miR‐140‐5p were obtained in published literature and were compared with the predicted results of this study.

## Materials and methods

### Mature sequences of miR‐140‐5p in various species

Mature sequences of miR‐140‐5p in various species were obtained in the miRBase database (http://mirbase.org/index.shtml).

### Target gene prediction of miR‐140‐5p

Identification of target genes is critical for characterizing the functions of miRNAs. In this study, miRanda (http://www.microrna.org/), TargetScan (http://www.targetscan.org/), RNAhybrid (https://bibiserv.cebitec.uni-bielefeld.de/rnahybrid/submission.html), and miRDB (http://www.mirdb.org/) databases were used to predict the target genes of miR‐140‐5p. To make our predicted target genes more convincible, only the target genes predicted by at least three databases were selected for further analyses.

### Database‐based GO and KEGG pathway enrichment analysis

Target mRNA of miR‐140‐5p supported by at least three databases were used for GO analysis to predict gene functions. Integration Discovery (DAVID) software, version 6.7 (http://david.abcC.ncifcrf.gov), was used to perform GO analysis to identify biological processes, cellular components, and molecular functions of these target genes. At the same time, the probable signaling pathways in which these target genes were enriched were analyzed by KEGG database (http://www.genome.jp/kegg/). The *P*‐value <0.05 was considered significant.

### Upstream TFs prediction of miR‐140‐5p

Human miR‐140‐5p precursor was obtained in the miRBase database and its 5000 bp upstream was defined as the miR‐140‐5p promoter. The TFs of miR‐140‐5p were predicted using MOODS‐python software (version 1.9.3) in JASPAR database (http://jaspar.binf.ku.dk/), which includes various vertebrate TFs. The *P*‐value <0.0001 was considered significant.

### Construction of the network for TF–miR‐140‐5p–mRNA

By merging the regulatory relationships between TFs and miR‐140‐5p, miR‐140‐5p and target genes, genes and genes (TF→miRNA, miRNA→gene and gene→gene), we constructed a comprehensive TF–miR‐140‐5p–mRNA regulatory network using Gephi software (release 0.8.1‐β, http://gephi.github.io/).

### Screening target genes and signaling pathways inhibited by miR‐140‐5p in published studies

To obtain downstream target genes and signaling pathways modulated by miR‐140‐5p in published studies, a comprehensive electronic search of Web of Science and PubMed databases was performed until April 20, 2017. The keyword ‘miR‐140‐5p’ in the titles or abstracts was used, and then, studies exploring the targets of miR‐140‐5p were collected.

## Results

### Mature sequences of miR‐140‐5p in various species

Mature sequences of miR‐140‐5p in various species were obtained in the miRBase database. The pre‐miR‐140‐5p was located at position 69933081 ~ 69933180 of chromosome 16, and the gene ID of human miR‐140‐5p was MIMAT0000431. As shown in Table 1, mature sequences of miR‐140‐5p were highly conserved in various species and human miR‐140‐5p was chosen for further analyses.

**Table 1:** Mature sequences of miR‐140‐5p in various species

ID	Mature name	Sequence
MIMAT0000151	mmu‐miR‐140‐5p	CAGUGGUUUUACCCUAUGGUAG
MIMAT0000431	hsa‐miR‐140‐5p	CAGUGGUUUUACCCUAUGGUAG
MIMAT0000573	rno‐miR‐140‐5p	CAGUGGUUUUACCCUAUGGUAG
MIMAT0001159	gga‐miR‐140‐5p	AGUGGUUUUACCCUAUGGUAG
MIMAT0001836	dre‐miR‐140‐5p	CAGUGGUUUUACCCUAUGGUAG
MIMAT0002143	ssc‐miR‐140‐5p	AGUGGUUUUACCCUAUGGUAG
MIMAT0006812	oan‐miR‐140‐5p	CAGUGGUUUUACCCUAUGGU
MIMAT0006197	mml‐miR‐140‐5p	CAGUGGUUUUACCCUAUGGUAG
MIMAT0012745	mdo‐miR‐140‐5p	CAGUGGUUUUACCCUAUGGUAG
MIMAT0012926	eca‐miR‐140‐5p	CAGUGGUUUUACCCUAUGGUAG
MIMAT0014557	tgu‐miR‐140‐5p	CAGUGGUUUUACCCUAUGGUAG
MIMAT0015763	ppy‐miR‐140‐5p	CAGUGGUUUUACCCUAUGGUAG
MIMAT0021765	aca‐miR‐140‐5p	CAGUGGUUUUACCCUAUGGU
MIMAT0022552	ola‐miR‐140‐5p	CAGUGGUUUUACCCUAUGGUAG
MIMAT0023767	cgr‐miR‐140‐5p	CAGUGGUUUUACCCUAUGGUAG
MIMAT0025434	pol‐miR‐140‐5p	CAGUGGUUUUACCCUAUGGUAG
MIMAT0026220	ccr‐miR‐140‐5p	CAGUGGUUUUACCCUAUGGUAG
MIMAT0032359	ssa‐miR‐140‐5p	CAGUGGUUUUACCCUAUGGUAG
MIMAT0035960	chi‐miR‐140‐5p	CAGUGGUUUUACCCUAUGGUAG
MIMAT0036560	tch‐miR‐140‐5p	CAGUGGUUUUACCCUAUGGUA
MIMAT0036719	oha‐miR‐140‐5p	CAGUGGUUUUACCCUAUGGUAG

### Prediction of target genes for miR‐140‐5p

**Figure 1:**
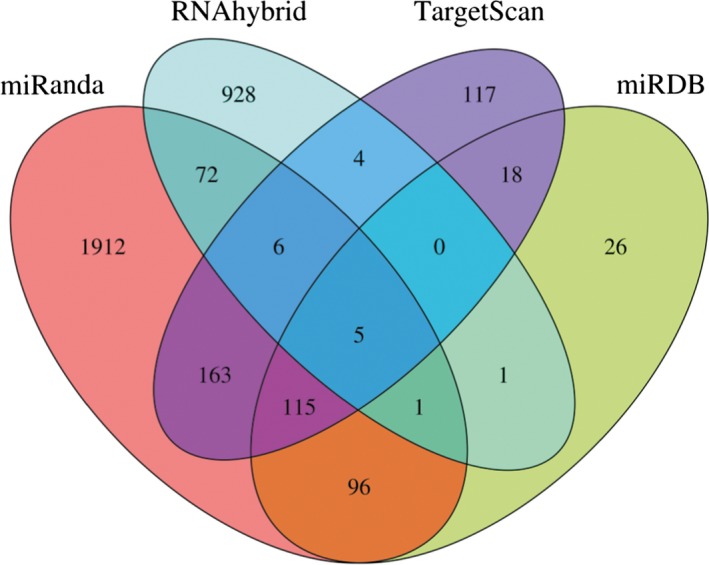
The number of predicted target genes of miR‐140‐5p.

As shown in Fig. 1, the number of predicted target genes of miR‐140‐5p in miRanda, TargetScan, RNAhybrid, and miRDB databases was 2370, 428, 1017, and 262, respectively. There were 482 target genes supported by at least two databases, 123 target genes predicted by at least three databases and five target genes supported by all four databases. The target genes of miR‐140‐5p predicted by at least three databases are listed in Table 2 and were used for further analyses.

**Table 2:** The target genes of miR‐140‐5p predicted by at least three databases

ABCA1	ACSL6	ADAM10	ADAMTS5	ADCY6	ANKFY1
ANKIB1	AP2B1	BACH1	BAZ2B	BCL9	BMP2
C1R	CADM3	CAND1	CAPN1	CCNYL1	CELF1
CORO2A	CREB	CTCF	CYTH2	DNM3	DOK4
DPP10	DPYSL2	EGR2	EIF4G2	ELAVL2	ENTPD5
EPB41L2	ERC2	FAM175B	FBN1	FCHO2	FES
FGF9	FLRT2	FOXP2	FYCO1	GNG5	GIT1
HAND2	HDAC4	HDAC7	HDGFRP3	HNRNPH3	HS2ST1
HSPA13	IGSF3	IPO7	JAG1	KAT2B	KBTBD2
KIF1B	KLF6	KLF9	KLK10	LAMC1	LHFPL2
LMNB1	LPHN2	LRAT	LRP4	LSM14B	LYSMD3
MARK1	MED13	MMD	MYCBP2	MYO10	NAA20
NAALADL2	NCKAP1	NCOA1	NCSTN	NFE2L2	NLK
NPL	NUCKS1	OSBPL6	PPPICC	PAFAH1B2	PDGFRA
PPTC7	PDE7A	PPP1R12A	PALM2‐AKAP2	RBM39	RFX7
RNF19A	RALA	RAB10	SEPT2	STRADB	SYS1
SLAIN1	SAMD4	SMOC2	SNX2	SRCAP	SHROOM3
SIAH1	SLC30A5	SLC38A2	TTYH3 ST5	TLR4	TTK
TJP1	TSSK2	TSPAN12	TSC22D2	TTYH2	TGFBR1
UBR5	UBR5	VEZF1	VEGFA	WNT1	WDFY3
YOD1	ZBTB10	ZNF800			

### GO enrichment analysis for predicted target genes of miR‐140‐5p

**Figure 2:**
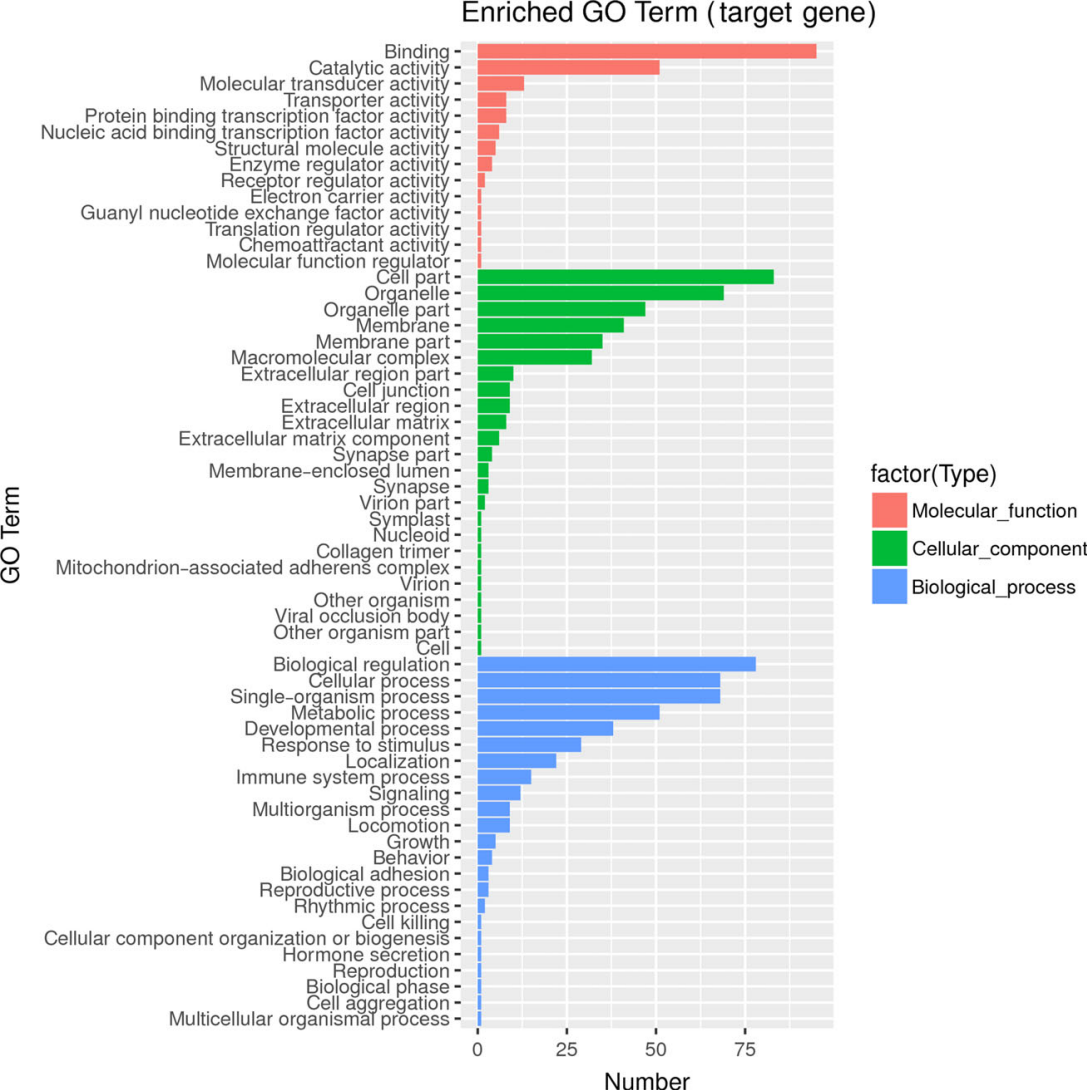
Gene ontology (GO) enrichment analysis for predicted target genes of miR‐140‐5p.

GO enrichment analysis was conducted for the target genes of miR‐140‐5p predicted by at least three databases. As shown in Table 3, the target genes of miR‐140‐5p were mainly located in basement membrane (*P *<* *0.05) and participated in the molecular functions of protein binding, activating transcription factor binding, ion binding, lipid binding, and so on (*P *<* *0.05). In addition, the target genes of miR‐140‐5p were involved in various biological processes, including biological regulation, metabolic process, cell communication, signal transduction, response to chemical stimulus, stem cell proliferation, cell surface receptor signaling pathway (*P *<* *0.05). Fig. 2 presents the number of target genes corresponding to each GO term.

**Table 3:** Gene ontology (GO) analysis for predicted target genes of miR‐140‐5p

ID	Term	*P*‐value	Genes annotated to the term
Biological processes			
GO:0050794	Regulation of cellular process	5.39E‐06	VEGFA| FGF9| PPP1CC|Pin1|HDAC7|PDGFRA|TGFBR1|ADAM10…
GO:0050789	Regulation of biological process	9.05E‐06	FGF9|BMP2|LAMC1|NUMBL|PDGFRA|PPP1CC||ADAM10|TLR4|TGFBR1…
GO:0007154	Cell communication	5.69E‐05	WNT1|PPP1CC|PDGFRA|TLR4|HDAC7|ADAM10| BMP2|TGFBR1…
GO:0023052	Signaling	6.14E‐05	PDGFRA|PPP1CC|FGF9|WNT1|TGFBR1|BMP2|ADAM10|JAG1|TLR4…
GO:0044763	Single‐organism cellular process	8.73E‐05	VEGFA|FGF9|LAMC1|BMP2|TLR4|WNT1|TGFBR1|PDGFRA|PPP1CC…
GO:0065007	Biological regulation	9.89E‐05	VEGFA|BMP2|TLR4|CREB|PPP1CC|PDGFRA|ADAM10|TGFBR1…
GO:0007165	Signal transduction	0.00011	PPP1CC|PDGFRA|WNT1|TGFBR1|FGF9|VEGFA|NCSTN|TLR4|ADAM10…
GO:0042221	Response to chemical stimulus	0.00048	NUMBL|PPP1CC|PDGFRA|VEGFA|LAMC1|TGFBR1|FGF9|BMP2|ADAM10|TLR4…
GO:0072089	Stem cell proliferation	0.00087	ACSL6|NUMBL|RAB10|HAND2|WNT1|BMP2…
GO:0007166	Cell surface receptor signaling pathway	0.00370	TLR4|WNT1|BMP2|ADAM10|NCSTN|JAG1|PPP1CC|PDGFRA|FGF9…
GO:0050896	Response to stimulus	0.01555	PPP1CC|PDGFRA|WNT1|CREB|TGFBR1|VEGFA||FGF9|BMP2|ADAM10|TLR4…
GO:0019538	Protein metabolic process	0.02054	CREB|PPP1CC|PDGFRA|NUMBL|TLR4|ADAM10|BMP2|KAT2B|NCSTN| TGFBR1…
GO:0006464	Cellular protein modification process	0.03073	HDAC4|CREB|ADAM10|TLR4|TGFBR1|PPP1CC|PDGFRA…
Molecular functions			
GO:0005515	Protein binding	2.53E‐07	TLR4|ADAM10|PDGFRA|WNT1|HDAC7|VEGFA|CREB|PPP1CC|TGFBR1|FGF9…
GO:0005488	Binding	0.00048	HDAC7|JAG|LMNB1|PDGFRA|ADAM10| TLR4|FGF9|KAT2B|TGFBR1…
GO:0033613	Activating transcription factor binding	0.00320	EGR2|NFE2L2|HDAC4|HDAC7|HAND2…
GO:0043167	Ion binding	0.00724	VEGFA|PPP1CC|ADAM10|PDGFRA|TGFBR1|HDAC4|FGF9|HDAC7…
GO:0008289	Lipid binding	0.04471	LAMC1|OSBPL6|FES|DNM3|MYO10|TLR4…
Cellular components			
GO:0005604	Basement membrane	0.04119	FGF9|PDGFRA|TLR4|VEGFA|SMOC2…

### KEGG pathway analysis for predicted target genes of miR‐140‐5p

**Figure 3:**
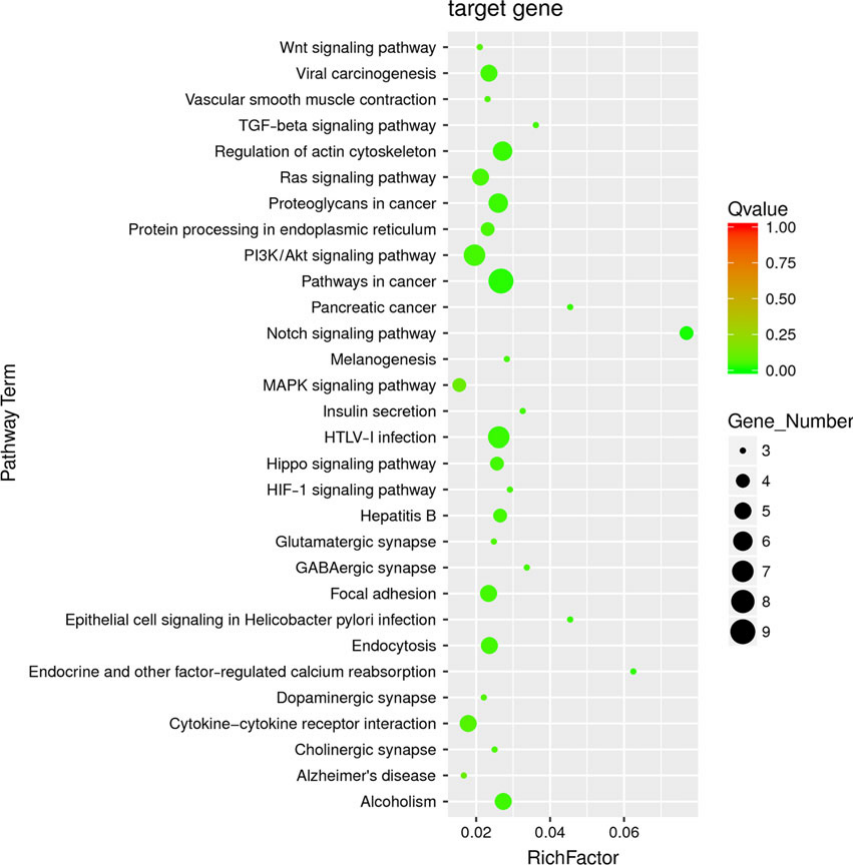
Kyoto Encyclopedia of Genes and Genomes (KEGG) pathway analysis for predicted target genes of miR‐140‐5p.

Enriched signaling pathways for the target genes of miR‐140‐5p identified by KEGG pathway analysis were ranked according to the *P*‐values. As shown in Table 4, the top rankings were related to Notch, cancer‐associated pathway, TGF‐beta, PI3K/Akt, HTLV infection, Hippo, HIF‐1, alcoholism signaling pathways, and so on (*P *<* *0.05); among them, Notch, TGF‐beta, PI3K/Akt, and Hippo signaling pathways were well known to be associated with the pathogenesis of PAH. Fig. 3 presents the rich factor, Q value, and gene number corresponding to each pathway term.

**Table 4:** Kyoto Encyclopedia of Genes and Genome (KEGG) pathway analysis for predicted target genes of miR‐140‐5p

Term	ID	Sample number	Background number	*P*‐value	Genes
Notch signaling pathway	hsa04330	4	52	0.006408	JAG1|ADAM10|KAT2B|NCSTN
Pathways in cancer	hsa05200	9	337	0.016384	FGF9|TGFBR1|VEGFA|SLC2A1|WNT1|BMP2|PDGFRA|LAMC1
Endocrine and other factor‐regulated calcium reabsorption	hsa04961	3	48	0.022347	AP2B1|ADCY6|DNM3
HTLV‐I infection	hsa05166	7	268	0.031935	TGFBR1|KAT2B|SLC2A1|EGR2|WNT1|PDGFRA|ADCY6
Regulation of actin cytoskeleton	hsa04810	6	221	0.031935	PPP1R12A|NCKAP1|FGF9|GIT1|PDGFRA|PPP1CC
Pancreatic cancer	hsa05212	3	66	0.031935	RALA|TGFBR1|VEGFA
Epithelial cell signaling in Helicobacter pylori infection	hsa05120	3	66	0.031935	TJP1|GIT1|ADAM10
Proteoglycans in cancer	hsa05205	6	231	0.033735	PPP1R12A|FGF9|VEGFA|WNT1|TLR4|PPP1CC
Adherence junction	hsa04520	3	74	0.037848	NLK|TJP1|TGFBR1
Alcoholism	hsa05034	5	183	0.038681	HDAC7|HDAC4|CREB3L1|GNG5|PPP1CC
PI3K‐Akt signaling pathway	hsa04151	7	358	0.045545	FGF9|VEGFA|PDGFRA|LAMC1|TLR4|CREB|GNG5
Focal adhesion	hsa04510	5	214	0.045545	PPP1R12A|VEGFA|PDGFRA|LAMC1|PPP1CC
Endocytosis	hsa04144	5	212	0.045545	AP2B1|TGFBR1|GIT1|PDGFRA|DNM3
Viral carcinogenesis	hsa05203	5	213	0.045545	HDAC7|HDAC4|KAT2B|EGR2|CREB3L1
Hepatitis B	hsa05161	4	151	0.045545	TGFBR1|EGR2|TLR4|CREB3L1
Insulin secretion	hsa04911	3	92	0.045545	SLC2A1|CREB3L1|ADCY6
GABAergic synapse	hsa04727	3	89	0.045545	SLC38A2|GNG5|ADCY6
TGF‐beta signaling pathway	hsa04350	3	83	0.045545	TGFBR1|SMAD4|BMP2
Gap junction	hsa04540	3	96	0.045545	TJP1|PDGFRA|ADCY6
Hippo signaling pathway	hsa04390	4	156	0.045565	TGFBR1|WNT1|BMP2|PPP1CC

### Prediction of upstream TFs for miR‐140‐5p and construction of TF–miR‐140‐5p–mRNA network

**Figure 4:**
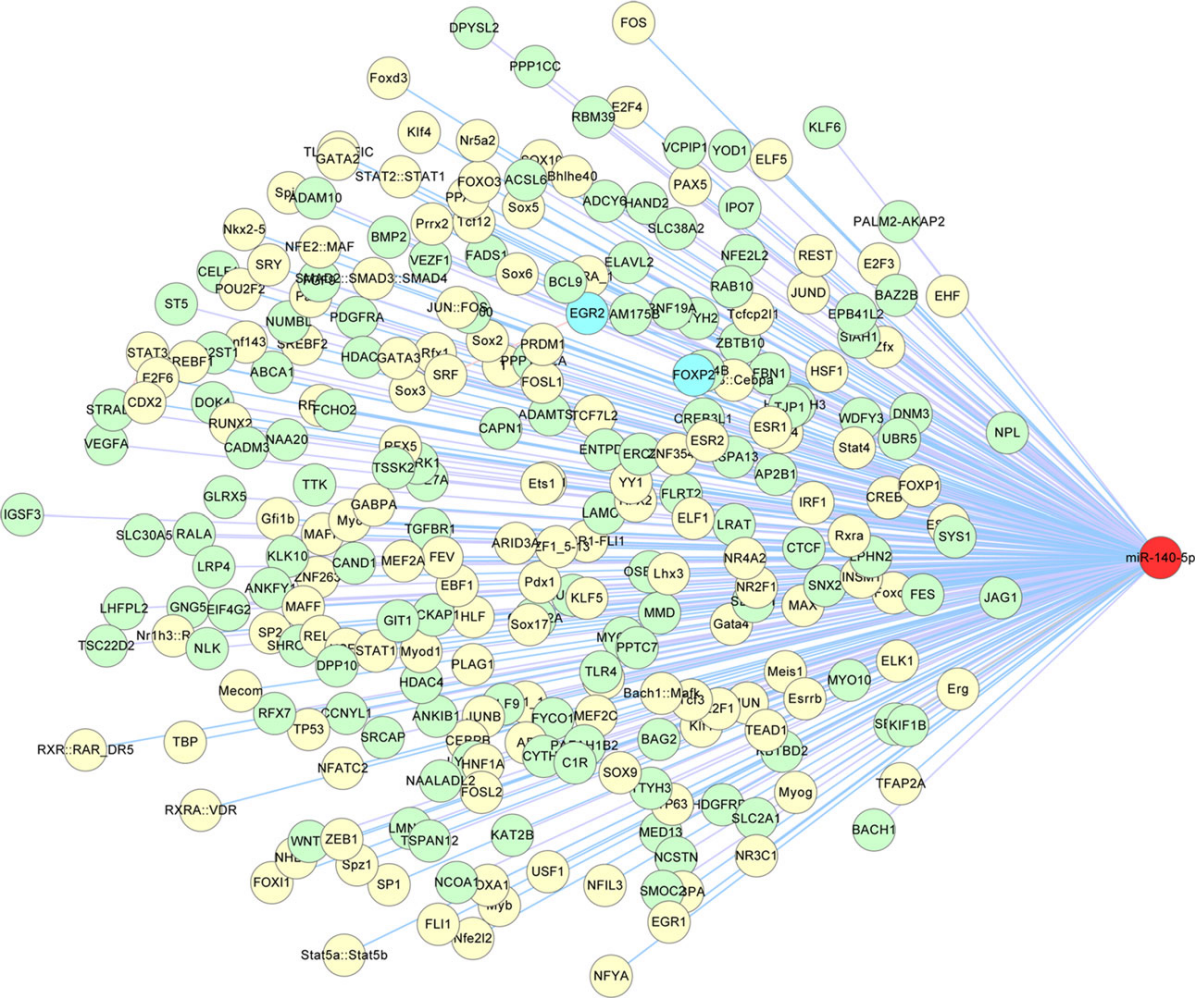
Regulatory network of TF–miR‐140‐5p–mRNA.

The number of predicted TFs for miR‐140‐5p with *P*‐value <0.0001 was 393. To reduce false‐positive results, TFs with a quality score (Q‐score) less than 10 were filtered. As shown in Table 5, the remaining TFs, including PAX5, FOXI1, IRF1, FOSL1, RUNX2, were chosen for further analyses. Finally, by merging the regulatory relationships between TFs and miR‐140‐5p, miR‐140‐5p and target genes, as well as genes and genes, we built a comprehensive TF–miR‐140‐5p–mRNA regulatory network, as shown in Fig. 4.

**Table 5:** Prediction of transcription factors and binding sites of miR‐140‐5p

Model ID	Model name	Hit position	Strand	Score	Predicted site sequence
MA0014.2	PAX5	95	−	10.5663	gtctcactctgttgcccat
MA0014.2	PAX5	3874	−	11.6915	gtcttgctctgttgcccag
MA0025.1	NFIL3	722	−	10.0393	TTCTTACATAA
MA0035.3	Gata1	3391	−	10.0718	acagataaaaa
MA0036.2	GATA2	3391	−	10.4087	acagataaaaattt
MA0041.1	Foxd3	4529	+	10.4011	ttttgtttgttt
MA0042.1	FOXI1	984	+	11.5926	GGATGTTTGTTT
MA0042.1	FOXI1	4529	+	10.3990	ttttgtttgttt
MA0046.1	HNF1A	4949	+	10.3282	agttaataatttta
MA0050.2	IRF1	3825	+	11.0065	tttttctttttcttttctttc
MA0050.2	IRF1	3840	+	12.4803	tctttctttcttttttttttt
MA0050.2	IRF1	3844	+	10.0776	tctttcttttttttttttttt
MA0062.2	GABPA	1506	+	10.0387	ccggaagtcga
MA0073.1	RREB1	1164	−	10.9028	TTTTGGTTGTTGTTTTGTTT
MA0073.1	RREB1	3734	+	10.2056	caacaaaacaaaacaaaaca
MA0471.1	E2F6	143	−	10.6410	tcttcccgcct
MA0477.1	FOSL1	4238	−	11.2229	cctgagtcacc
MA0478.1	FOSL2	4239	−	10.3145	ctgagtcacct
MA0481.1	FOXP1	3756	+	10.2195	acaaaaaaaacacaa
MA0481.1	FOXP1	4018	−	10.3465	ttttgtttttttagt
MA0490.1	JUNB	4239	−	10.6046	ctgagtcacct
MA0491.1	JUND	2362	+	10.0256	GAAAATGATATCACA
MA0493.1	Klf1	4812	+	10.548	caccacaccca
MA0511.1	RUNX2	3813	+	11.453	tgtgtatgtggtttt
MA0515.1	Sox6	3772	−	10.2529	gaaacaatgg
MA0595.1	SREBF1	2000	−	10.1772	gtggcgtgat

### Screening target genes and signaling pathways modulated by miR‐140‐5p in published studies

A comprehensive electronic search of Web of Science and PubMed databases was performed until April 20, 2017, to obtain target genes and signaling pathways modulated by miR‐140‐5p in published studies. Finally, a total of 26 papers including 23 target genes and seven signaling pathways inhibited by miR‐140‐5p were obtained; most of them focus on the functions of miR‐140‐5p suppressing tumor growth, migration, and invasion in various tumor tissues and cells. Two recent studies have found that SMURF1 and Dumt1 are direct target genes of miR‐140‐5p in pulmonary arterial smooth muscle cells (PASMCs) and are involved in the pathogenesis of PAH. The details are shown in Table 6.

**Table 6:** Target genes and signaling pathways modulated by miR‐140‐5p in published studies. NA, not available; HCC, hepatocellular carcinoma; T‐ALL, T‐cell acute lymphoblastic leukemia; Th1, T helper type 1; HSCC, hypopharyngeal squamous cell carcinoma; EPCs, endothelial progenitor cells; PH, pulmonary hypertension; HUVECs, human umbilical vein endothelial cells; BTC, biliary tract cancer; TSPCs, tendon stem/progenitor cells; LLC, Lewis lung cancer cells; MSCs, mesenchymal stem cells; TSCC, tongue squamous cell carcinoma

Author (Year)	Target genes	Inhibited pathways	Associated functions	Cell or tissue types
Hu (2017)	VEGFA	NA	Inhibit cell proliferation and invasion, promote apoptosis	Glioma tissues and cells
Meng (2017)	HMGN5	NA	Decrease cell resistance to chemotherapy	Osteosarcoma tissues and cells
Yan (2017)	Pin1	Pin1‐dependent cancer pathway	Suppress tumor growth	HCC tissues and cells
Correia (2016)	TAL1	NA	Suppress tumor growth	T‐ALL cells
Guan (2016)	STAT1	NA	Suppress Th1 cell differentiation	Th1 cells
Jing (2016)	ADAM10	Notch1 signaling pathway	Suppress tumor migration and invasion	HSCC tissues and cells
Liu (2016)	HDAC7	NA	Protect EPCs	EPCs
Lv (2016)	Slug	NA	Inhibit cell migration and invasion	HCC tissues
Rothman (2016)	SMURF1	BMP signaling pathway	Inhibit cell proliferation, migration, and PH development	PASMCs, rat PH models
Su (2016)	IGF2BP1	NA	Decrease cell proliferation, migration, and invasion	Cervical cancer cells and tissues
SUN (2016)	VEGFA	NA	Decrease cell proliferation, migration, and tube formation	HUVECs
Wei (2016)	IP3k2	IP3 signaling pathway	Promote chemotherapy‐induced autophagy	Human osteosarcoma cells
Yu (2016)	Septin 2	NA	Suppress cell proliferation and colony formation	BTC tissues and cells
Zhang (2016)	Dnmt1	NA	Inhibit cell proliferation, promote cell apoptosis	Human PH tissues, human PASMCs
Barter (2015)	FZD6	Wnt signaling pathway	Promote chondrogenic differentiation	Mesenchymal stem cells
Chen (2015)	Pin1	NA	Promote cell senescence	TSPCs
Lan (2015)	PDGFRA	NA	Inhibit cancer growth	Human ovarian cancer tissues and cells
Zhai (2015)	Smad2	TGF‐β signaling pathway	Decrease cell invasion and proliferation	Colorectal cancer stem cells
Zhang (2015)	VEGFA	NA	Inhibit tumor progression	Colorectal cancer tissues and cells
Zhang (2015)	TGFBR1	TGF‐β signaling pathway	Regulate adipocyte differentiation	Bone marrow stromal cells
Li (2014)	MMD	ERK signaling pathway	Inhibit cell proliferation	LLCs
Hwang (2014)	BMP2	BMP signaling pathway	Suppress osteogenesis	Human MSCs
Karlsen (2014)	RALA	NA	Stimulate chondrogenesis	MSCs
Yang (2014)	ADAM10, LAMC1, HDAC7	NA	Suppress migration and invasion	TSCC tissues and cells
Shi (2013)	FoxP2	NA	Impair dendritic development and vocal learning	Zebra finch brain tissues
Yang (2013)	TGFBR1, FGF9	TGF‐β and ERK signaling pathway	Suppress cell proliferation and tumor metastasis	HCC tissues and cells

## Discussion

Pulmonary arterial hypertension is a chronic life‐threatening condition requiring long‐term management 13, and its available therapies are limited 6. There is a clear and urgent need for new therapeutic options based on deeply exploring the pathogenesis of PAH. Previous studies have indicated that miR‐140‐5p is dramatically downregulated, which in turn causes the development of a variety of cancers by the loss of suppressing tumor cell migration and growth 14, 15, 16, 17. miR‐140‐5p has been recently found to be reduced in both PAH patients and MCT‐induced PAH models in rat 11, 12. However, the downstream targets regulated by miR‐140‐5p contributing to the development of PAH remain largely unknown.

In this study, we found that the target genes of miR‐140‐5p were enriched in many biological processes, such as biological regulation, metabolic process, cell communication, signal transduction, response to chemical stimulus, stem cell proliferation, cell surface receptor signaling pathway. In KEGG pathway analysis, the target genes of miR‐140‐5p were mainly located in Notch, TGF‐beta, PI3K/Akt, and Hippo signaling pathways. According to the TF–miRNA–mRNA network, the important genes potentially regulated by miR‐140‐5p included PPI, TGF‐betaR1, smad4, JAG1, ADAM10, FGF9, PDGFRA, VEGFA, TLR4, LAMC1, CREB, and the upstream TFs, which might regulate miR‐140‐5p expression including TAX5, FOXI, IRF1, GATA6, RUNX2. After thoroughly reviewing published literature, we found that 23 target genes and seven signaling pathways were truly inhibited by miR‐140‐5p in various tissues or cells; most of these downstream targets were in accordance with our present prediction.

Several studies have shown that activation of Notch3 pathway is involved in the pathogenesis of PAH 18, 19. We have previously shown that activation of Notch3 promotes PASMC proliferation and inhibition of Notch3 pathway prevents monocrotaline‐induced development of PAH in rat 20, 21. JAG1 and ADAM10 are indispensable components of Notch signaling pathway, which were predicted as downstream targets of miR‐140‐5p in our analysis, suggesting that lack of miR‐140‐5p might promote the development of PAH by upregulation of JAG1 and ADAM10 genes and therefore activation of Notch3 cascade. In addition, activation of TGF‐beta1/Smad4 signaling promotes a proliferative PASMC phenotype and induces PAH in rat 22, 23. We found that TGF‐betaR1 and smad4 were possible downstream targets of miR‐140‐5p, reduction in miR‐140‐5p in PAH might stimulate TGF‐beta1/Smad4 pathway by upregulating TGF‐betaR1 and smad4. Previous studies have demonstrated that PDGF, TLR4, VEGFA, and FGF contribute to the pathogenesis of PAH via activating various signaling pathways, especially PI3K/Akt cascade 24, 25, 26, 27, 28. CREB, an important transcription factor lying downstream of PI3K/Akt pathway, mediates the partial functions of PI3K/Akt 29. In our analysis, PDGF, TLR4, VEGFA, FGF, and CREB were positively predicted as downstream targets of miR‐140‐5p, implying that miR‐140‐5p negatively regulates the functions of PI3K/Akt cascade by targeting FGF9, PDGFRA, VEGFA, TLR4, or CREB gene. Recent studies have also shown that Hippo signaling is associated with the development of PAH, which can be activated by PPI 30, 31. Our present results suggested that PPI was a direct target gene of miR‐140‐5p and might mediate miR‐140‐5p regulation of Hippo signaling.

Our predicted network provided potential target genes and relevant signaling pathways that might be modulated by miR‐140‐5p contribution to the development of PAH. Several targets and pathways predicted in our analysis, such as TGF‐betaR1, ADAM10, FGF9, PDGFRA, VEGFA and Notch, PI3K/Akt, TGF‐beta cascades, have been demonstrated to mediate the effects of miR‐140‐5p on antiproliferation and prodifferentiation in several cell types in published studies 16, 17, 32, 33. While the other targets predicted in our study, including PPI, smad4, JAG1, LAMC1, TLR4, and CREB as well as Hippo signaling pathway, have not been confirmed in the published literature, they still need further verification *in vivo* and *in vitro*.

## Author contributions

ML and FL designed the study; WS, YW, LC, and QW analyzed and interpreted the data; WF, XY, QZ, and JW organized the results; FL wrote the manuscript.
